# Ubiquitin regulates dissociation of DNA repair factors from chromatin

**DOI:** 10.18632/oncotarget.4410

**Published:** 2015-06-10

**Authors:** Petra Beli, Stephen P. Jackson

**Affiliations:** Institute of Molecular Biology (IMB), Mainz, Germany

Involvement of the ubiquitin system in DNA repair and DNA damage signaling has been extensively studied during the last decade. Dynamic modification of proteins with ubiquitin after DNA damage has been shown to play important roles in virtually all DNA repair pathways [1]. In this regard, protein ubiquitylation has most frequently been associated with the recruitment of DNA repair factors – many containing ubiquitin-binding domains – to DNA lesions and/or flanking chromatin. A prominent example of this is monoubiquitylation of the replication clamp PCNA by the ubiquitin ligase RAD18 that leads to recruitment of error-prone DNA polymerases and ensuing translesion DNA synthesis. In addition, ubiquitylation of histone variant H2AX after DNA damage by the ubiquitin ligases RNF8 and RNF168 promotes the repair and signaling of DNA double strand breaks (DSBs) and leads to recruitment of downstream repair factors such as BRCA1 and 53BP1 [1].

Ubiquitin-dependent extraction of chromatin-associated proteins from repair sites is also now emerging as an important mechanism for maintaining chromatin and genome integrity. In this regard, we have recently shown that the Ku heterodimer, which mediates DSB repair by non-homologous end joining (NHEJ), is extracted from chromatin in an ubiquitin-dependent process [2]. NHEJ is the predominant DSB repair pathway in human cells, and is initiated by the binding of Ku to DNA ends. Ku forms a stable ring structure encircling DNA, and together with factors such as DNA-PKcs and PAXX, forms an assembly that mediates DSB repair by the DNA ligase IV/XRCC4/XLF complex. Although components of the NHEJ pathway have been well characterized, the mechanisms that promote their dissociation from repair sites remain poorly understood. It has long been recognized that this dissociation is important because prolonged binding of Ku to chromatin after DSB repair would interfere with transcription and replication, thereby posing serious threats to the cell.

We have found that the ubiquitin-like protein NEDD8 is recruited to DSB sites in human cells together with various components of the NEDD8-conjugation machinery [2]. Moreover, we established that NEDD8 conjugation actively occurs at such sites, and that depleting neddylation components impairs cell survival after DSB induction. Strikingly, through using NEDD8-activating enzyme inhibitor MLN4924, we established that neddylation does not appear to markedly affect DSB repair but rather promotes the removal of Ku and other NHEJ components from chromatin after repair has taken place.

Previous work has established that a key role for neddylation is to activate a conserved family of ubiquitin ligases Cullin-RING ligases (CRLs) that regulate diverse cellular processes by mediating ubiquitylation and subsequent degradation of substrate proteins through the ubiquitin-proteasome system. Modification of CRLs with NEDD8 increases CRL ubiquitylation activity through conformational changes that optimize ubiquitin transfer to target proteins. In accord with this, through proteomics studies, we established that neddylation mediates ubiquitylation of Ku after DNA damage and that this is connected to Ku associating with components of the proteasome and the segregase/unfoldase protein VCP/p97. As VCP has established roles in remodeling and dismantling protein complexes, these findings lead to a model in which NEDD8-dependent ubiquitylation of Ku after/during DNA repair promotes VCP-mediated extraction of Ku from chromatin, possibly followed by proteasome-dependent Ku degradation. This study thus delivers insights into the mechanisms that underlie Ku dissociation from chromatin after repair and highlights a pervasive role of ubiquitin in the dynamic assembly and disassembly of protein complexes on chromatin after DNA damage.

In addition to our work, several other recent studies have pointed to important, but so far relatively unexplored roles of ubiquitin in the timely removal and/or degradation of DNA repair factors and chromatin-associated proteins during and/or after DNA repair. For instance, ubiquitin and VCP have been shown to promote the removal of the Polycomb protein L3MBTL1 from chromatin, thereby facilitating recruitment of 53BP1 to DSB sites [3]. Furthermore, in response to ultra-violet light-induced DNA damage, ubiquitin regulates the removal of the nucleotide excision repair factor XPC from DNA lesions, and inhibition of this process causes DNA repair defects and genomic instability [4]. In addition, ubiquitin-dependent removal of DNA polymerase eta plays an important role in the switch from error-prone to replicative DNA polymerase during translesion DNA synthesis [5, 6].

Increasing knowledge about the involvement of the ubiquitin system in DNA damage repair and signaling has opened up novel opportunities for therapeutic interventions. For instance, inhibiting ubiquitin ligases or deubiquitylating enzymes with DNA repair connections might selectively kill cancer cells with dependencies on specific DNA repair pathways and/or associated processes. Furthermore, it will be interesting to test neddylation inhibitors such as MLN4924 in these settings, as well as seeing if such inhibitors display synergy with radiotherapy or DSB-inducing chemotherapeutic agents.
